# Siamese Neural Network for Speech-Based Depression Classification and Severity Assessment

**DOI:** 10.1007/s41666-024-00175-4

**Published:** 2024-10-03

**Authors:** Stavros Ntalampiras, Wen Qi

**Affiliations:** 1 Department of Computer Science, University of Milan, 20135 via Celoria 18, Milan, Italy; 2 School of Future Technology, South China University of Technology, 510641 Wushan Road 381, Guangzhou, China

**Keywords:** Medical acoustics, Audio pattern recognition, Representation learning, Depression, Affective computing, Explainable AI, Interpretable AI

## Abstract

The evaluation of an individual’s mental health and behavioral functioning, known as psychological assessment, is generally conducted by a mental health professional. This process aids in diagnosing mental health conditions, identifying suitable treatment options, and assessing progress during treatment. Currently, national health systems are unable to cope with the constantly growing demand for such services. To address and expedite the diagnosis process, this study suggests an AI-powered tool capable of delivering understandable predictions through the automated processing of the captured speech signals. To this end, we employed a Siamese neural network (SNN) elaborating on standardized speech representations free of domain expert knowledge. Such an SNN-based framework is able to address multiple downstream tasks using the same latent representation. Interestingly, it has been applied both for classifying speech depression as well as assessing its severity. After extensive experiments on a publicly available dataset following a standardized protocol, it is shown to significantly outperform the state of the art with respect to both tasks. Last but not least, the present solution offers interpretable predictions, while being able to meaningfully interact with the medical experts.

## Introduction

Inarguably, a considerable segment of the population experiences mental health disorders, leading to a notable decline in their overall quality of life [1]. Meanwhile, such diseases are connected to a significant economic impact, exceeding that of cancer, cardiovascular, and respiratory diseases [2]. Nevertheless, governmental budgets are generally more substantial for diseases other than mental illnesses. Currently, the diagnosis of these conditions primarily relies on reports generated either by the individuals themselves through self-assessment or by their caregivers. Despite the fact that such reports are often biased, they might offer important information during both diagnosis and treatment processes [3]. There is a crucial requirement to standardize the diagnosis process and ensure its ongoing accessibility, allowing individuals to receive regular treatment and evidence-based therapy. This is particularly important in areas where psychological services are scarce or nonexistent. Implementing periodic and methodical assessments would notably enhance therapeutic outcomes, thereby reducing the negative consequences associated with mental health challenges. The diagnostic process is heavily reliant on the presence of proficient professionals with specialized knowledge in the specific mental health condition. As a result, it becomes a time-consuming procedure associated with a disproportionately high cost. An alternative diagnostic approach addressing these limitations revolves around the creation of audio pattern recognition systems [4–6]. Among the diverse application of such systems, they are able to process speech signals and make inferences regarding an individual’s health status [7]. It is crucial to emphasize that the intention is not to substitute the expertise of a medical professional but to establish a supplementary tool capable of providing an initial diagnosis and potentially speeding up the evaluation process.

Within the realm of mental disorders, a considerable amount of studies have focused on utilizing speech processing to identify and detect signs of depression [8, 9]. In that context, both manually crafted and automatically learned features, along with the use of generative and discriminative classifiers, have been applied [7, 10–12]. This work does not encompass a systematic review, and readers interested in such a review are encouraged to refer to the comprehensive survey provided in [3]. Through a comprehensive examination of the related papers, several noteworthy observations arise concerning the current gaps: (a) poor data availability: there is not only a limited amount of publicly available datasets, but their size, in general, is restrictive. Nonetheless, there are works that employed deep architectures despite the curse of overfitting which may limit their generalization ability. On top of that, a standardized experimental protocol, enabling reproducible research and facilitating reliable comparisons of various approaches, is currently lacking. (b) dependence on domain knowledge: most of the current studies rely on a thorough understanding of problem specifications and the available dataset to appropriately craft the features used in the analysis, and (c) the research community has not extensively delved into the interpretation of model predictions, let alone interaction with the medical personnel, which might be connected with the limited use and acceptance of such AI-based tools and methodologies by such experts [13, 14].

Motivated by these considerations, this work introduces an SNN-based learning framework able to learn generic speech representations tightly coupled to the two principal problems in mental health assessment, i.e., depression classification and severity assessment. Interestingly, such a learning paradigm aims at uncovering universal representations from speech data, which can be subsequently exploited to address various related tasks. To the best of our knowledge, this is the first time that a representation learnt from an SNN is employed to address tasks of diverse requirements. At the same time, this work develops a suitable *k-medoids algorithm* which is employed for regression and outperforms the state of the art solutions.

The present article develops and examines SNN-based representations approaching different types of problems, i.e., classification and regression, where the aim is not only to identify but also to assess the severity of depression in speech segments. In addition, this work introduces the following novel points: (a) it employs a standardized feature set free of domain knowledge, (b) it learns and thoroughly applies a single representation to problems of diverse characteristics, (c) it introduces SNN for regression in the audio pattern recognition domain, (d) it is able to operate in limited and potentially imbalanced data environments, and (e) it offers both a clear interpretation of the decisions and the possibility to meaningfully interact with medical experts.

The cornerstone of the proposed framework is a Siamese neural network able to learn representations existing in standardized log-Mel spectrograms extracted from the available data (upstream task). Such features are then employed to address two downstream tasks of diverse requirements. More specifically, classification is based on analogies between the unknown speech segments and a suitably selected support set, while regression is carried out via a *k*-medoids scheme elaborating on the distances between the learnt representations. During the experimental phase, we employed the Hungarian Depressed Speech Database (DEPISDA) [15] which is a unique corpus including not only the result of the professional diagnosis but the corresponding severity of depression following the Hamilton Rating Scale for Depression (HAMD) score [16]. On top of that, the dataset comes with a predefined experimental protocol adopting the leave-one-subject-out division scheme, thus permitting a straightforward comparison between different approaches.

## Problem Formalization

Let us assume the availability of an annotated dataset including monophonic speech samples representing two mental states, i.e., class dictionary $\mathcal {D}$={*Healthy*, *Depressed*} along with the associated HAMD scores. Thus, two different but related problems arise, i.e., automatic classification of the mental state as well as prediction of its severity in terms of HAMD score. Following the related literature [15, 17, 18], we assume that a single patient may be characterized by one specific mental state and severity level throughout the entire duration of a given speech recording.

In addition, this article does not assume the existence of subject-specific knowledge beforehand. The primary aim is to predict (a) the mental state conveyed in novel speech segments, and (b) the respective HAMD score, while considering a subject-independent experimental protocol.

**Fig. 1:**
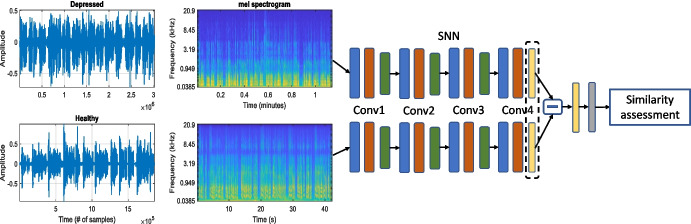
The topology of the proposed mental health assessment system using a Siamese neural network topology

## SNN-Based Representation Learning

This section describes the SNN-based framework designed to produce representations for addressing the prediction and assessment of mental health state. The respective block diagram is shown in Fig. 1. The framework relies on similarities existing in segments belonging to a single recording as well as dissimilarities between different recordings. As such, speech signals are processed in pairs, where the first step is common and aims at extracting a time-frequency representation. Following the research in audio pattern recognition and affective computing [19, 20], we rely on log-Mel spectrograms, i.e., speech signals are represented by 64 equal-width log-energies, the overlap of which is dictated by the Mel filterbank. The traditional extraction approach is adhered to, encompassing the calculation of the short-time Fourier transform. Mel spectrograms have demonstrated their ability to accentuate components that significantly contribute to human auditory perception.

Drawing upon the discoveries from our previous research [21, 22], we crafted specialized SNN learning representations able to address the specific problem. SNNs have proven to be effective in encoding relationships, particularly similarities and dissimilarities, among diverse audio signals. Consequently, they can be utilized for classification and/or regression purposes. An SNN comprises two neural networks, commonly referred to as twins, connected to a joint endpoint while processing distinct input signals [23]. The terminal point computes a predetermined distance metric by utilizing the abstract representations of the signals fed to each neural network. Since the endpoint is shared, the neural networks strive to optimize a common objective function. Consequently, the weights of each neural network’s layer are interconnected, resulting in closely aligned high-level representations for similar input signals. Notably, the architectures of the neural networks exhibit symmetry, indicating that both the networks themselves and their input configurations can be reversed, yet the output metric remains consistent. This symmetry underscores the robustness and flexibility of the model, allowing for interchangeable processing of inputs without affecting the final output calculation.

As the SNN undergoes training with pairs of time-frequency representations denoting similarities/dissimilarities, it inherently addresses the challenge of class imbalance characterizing the current task. This occurs when a well-evened set of both similar and dissimilar input pairs is utilized in the training process. The SNN’s ability to learn from such a diverse set of pairs ensures a more robust and equitable representation learning, enhancing its capacity to handle real-world scenarios with varying degrees of similarities/dissimilarities among audio signals.

**Algorithm 1:**
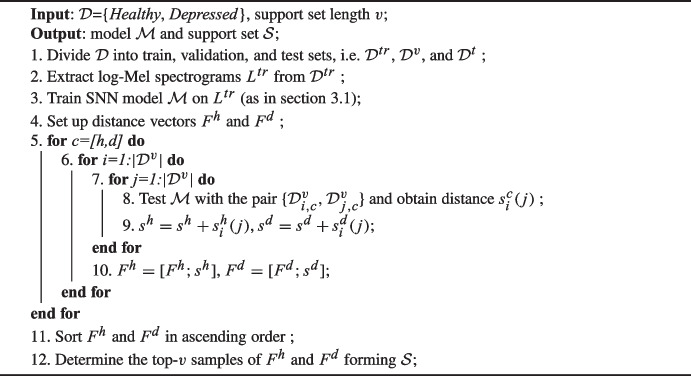
The algorithm which provides $\mathcal {S}$ ($|\bullet |$ denotes the cardinality operator).

### Model Architecture and Parameterization

Considering the efficacy of Convolutional Neural Networks (CNNs) in audio pattern recognition tasks [24–26], we construct each twin with a sequence of layered convolutional networks, each coupled with a corresponding max-pooling layer as illustrated in Fig. 1. It should be stressed out that such convolutional structures have the potential to emphasize local patterns within the bidimensional plane.

Every hidden unit within the network is equipped with information representing a delimited segment of the input signal, known as *receptive field*. The weights of the unit contribute to the creation of feature maps that specifically capture the distinctive attributes associated with that particular field. In essence, this design enables each hidden unit to focus on and process localized information, enhancing the network’s ability to discern and extract meaningful features from different regions of the input signal. Due to the potential vastness of the resulting dimensionality, pooling layers are integrated to selectively retain the maximum value within a designated zone.

After optimizing the hyperparameters, a phase which involved tuning parameters such as the number of layers, kernel and stride size, and learning rate, the SNN is configured with four convolutional layers. As depicted in Fig. 1, the initial three layers are followed by rectified linear unit (ReLU, activation function $f(x)=max(0,x)$) activations and subsequent max-pooling layers.

The ultimate layer of the network is a fully connected one, where the SNN calculates the distance between the outputs of the twin components using the binary cross-entropy loss. To ensure consistency and facilitate interpretation, this computed loss is then normalized to fall within the [0,1] range through the application of a sigmoid function characterizing the relationship between the inputs of the given pair. Such a loss value may be thresholded in order to decide upon a similar or dissimilar relationship.

It’s worth noting that the filters in the convolutional layers vary in size, with a consistent stride of one. Furthermore, the max-pooling layers employ kernels sized $2\times 2$, with a stride set to two. The learning process adheres to the traditional form of the back-propagation algorithm, wherein the update of weights for each twin is determined by summing gradients. Moreover, the batch size is set to 50, and the learning rate is 6e-5. The weights and biases are initialized using narrow normal distributions characterized by parameters $\mu =0$ and $\sigma =0.01$. The training process is set to undergo a maximum of 3000 iterations, incorporating the early stopping criterion. Finally, the representation learned by the last layer of each twin during the upstream task is used to address the two downstream tasks associated with mental health assessment, i.e., depression classification and evaluation of its severity.

## Downstream Task 1: Classification of Depression

This section analyzes the way the learned representations are used for classification, which is based on quantifying the similarities/dissimilarities existing between known and unknown speech segments. In the following, we describe the process to suitably select the known segments forming the support set denoted as $\mathcal {S}$ along with the classification process.

### Support Set Formation

After constructing the SNN-producing speech representations, the next step for utilizing them in classification involves the selection of a set of samples facilitating the evaluation of similarities/dissimilarities with each class. As such, unknown speech is going to be classified as depressed/healthy based on the analogies existing between itself and the support set. This process plays a principal role in facilitating the decision-making mechanism, as highlighted in [27, 28].

In this context, we propose an innovative approach for establishing the support set, denoted as $\mathcal {S}$. Our method, explicated in Algorithm 1, adopts a sample-wise similarity strategy. Essentially, this algorithm identifies samples that maximize the intra-class similarity on the available dataset. These selected samples contribute to the construction of the support set $\mathcal {S}$, thereby enhancing the system’s ability to make informed and accurate decisions in the classification process.

Algorithm 1 takes as input a dataset $\mathcal {D}$ annotated with classes *healthy* and *depression*, along with the user-defined parameter *v* representing the length of the support set. Its outputs include model $\mathcal {M}$ and support set $\mathcal {S}$ which is evenly spread across the existing classes.

Initially, the algorithm splits $\mathcal {D}$ into train, validation, and test sets, i.e., $\mathcal {D}^{tr}$, $\mathcal {D}^{v}$, and $\mathcal {D}^{t}$ (line 1, Algorithm 1). Subsequently, is computes the log-Mel spectrograms $L^{tr}$ from $\mathcal {D}^{tr}$ and trains the SNN model $\mathcal {M}$ (lines 2–3, Algorithm 1) as explained in Sect. 3. After setting up the distance vectors ($F^h$ and $F^d$) for each class (line 4, Algorithm 1), the algorithm performs a nested for loop, iterating through each class, and for each class, the corresponding samples are provided to the trained model $\mathcal {M}$. This process aims at populating both vectors $F^h$ and $F^d$ with the distances computed for each class. Following that, the sample-wise distances are summed and then incorporated in $F^h$ and $F^d$, which are then arranged in ascending order (lines 9–11, Algorithm 1). At the final stage, the support set is formed by the top-*v* samples of $F^h$ and $F^d$ (line 10, Algorithm 1).

### Classification of Unknown Speech Segments

In this section, the process for categorizing novel speech segments is outlined. Once model $\mathcal {M}$ and support set $\mathcal {S}$ are learned, the novel segment undergoes comparison with those existing in $\mathcal {S}$ utilizing $\mathcal {M}$, and the class with the highest similarity is determined as the winner. The detailed steps are provided in Algorithm 2

**Algorithm 2:**
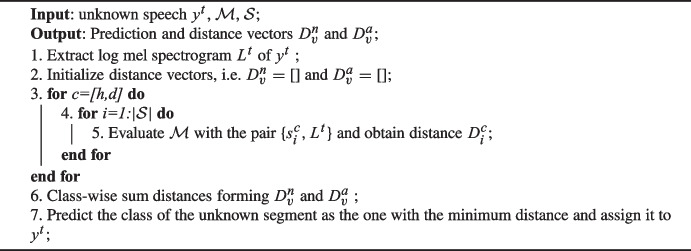
The proposed algorithm for the classification of unknown speech segments.

The algorithm takes the test speech signal $y^t$, along with $\mathcal {M}$ and $\mathcal {S}$, as inputs. The outputs consist of the prediction and the distance vectors $D_v^n$ and $D_v^a$. In the initial stage, the algorithm retrieves the log-Mel spectrogram $L^t$ that characterizes $y^t$ by employing the procedure detailed in Sect. 3 (line 1, Algorithm 2)

Following the initialization of distance vectors $D_v^n$ and $D_v^a$ (line 2, Algorithm‘2 the algorithm executes a nested for loop across the classes and components within $\mathcal {S}$. Within this loop, $\mathcal {M}$ is invoked with the pair ${s_i^{c},L^t}$ to acquire the distance value $D_i^c$ (lines 3–5, Algorithm 2.

Finally, the algorithm aggregates the distances for each class (line 6, Algorithm 2, and the predicted class is determined as the one with the minimum value (line 7, Algorithm 2.

## Downstream Task 2: Depression Severity Assessment

The second downstream task is concerned with the prediction of the depression’s severity as expressed by the HAMD score. The objective of the regression algorithm introduced is to pinpoint feature vectors with closely located HAMD scores. To attain this objective, we employ the *k-medoids algorithm* [29], which is part of the *k*-means clustering algorithms family. In contrast to the traditional *k*-means, *k*-medoids have the capability to integrate general pairwise similarity metrics, thereby reducing the impact of outliers [29]. Interestingly, such an approach is able to operate in a limited data availability environment since it is based on previously learned representations without building particularly complex models.

In our particular scenario, the components considered in the *k*-medoids algorithm are the log-Mel spectrograms. For the pairwise dissimilarity measure in the *k*-medoids algorithm, we propose to use the distance as provided by contrasting the representations learned by the SNN.

Let us denote $L^i$ and $L^j$ the log-Mel spectrograms extracted from two speech segments $y_i^t$ and $y_j^t$, respectively. The distance $d_{i,j}$ is given by the following formula:

1 $$\begin{aligned} d_{i,j}=\mathcal {M}(L^i,L^j). \end{aligned}$$

It is evident that lower values of $d_{i,j}$ indicate closer proximity between the respective speech segments in the SNN’s distance space. Importantly, $d_{i,j}$ satisfies the symmetry property, i.e., $d_{i,j}=d_{j,i}$.

The proposed procedure involves the identification of the *k* log-Mel spectrograms that exhibit the highest similarity to the unknown one $L^u$. The aim is to detect the *k* feature matrices in the available dataset that bear the highest resemblance to the novel one. Let us assume that their HAMD scores are $\xi _1,\dots ,\xi _k$. Based on these values, the unknown HAMD score is predicted based on the following formula:

$$\xi _u=\frac{1}{k}\sum \limits _{k=1}^{n}\xi _k$$.

In the proposed depression severity prediction methodology, the implementation of the *k*-medoids algorithm is based on the Partitioning Around Medoids [30]. The upcoming section outlines the experimental setup and evaluates the results obtained.

## Experimental Set-Up and Results

This section thoroughly explains the (a) employed dataset and suitable figures of merit, (b) parameterization of the proposed approach, and (c) experimental results, analysis, and comparison with the state of the art.

### The Dataset

In this study, we employed the Hungarian Depressed Speech Database (DEPISDA) [15], which comprises speech samples from 218 Hungarian individuals (144 females and 74 males), including both depressed and healthy subjects. Interestingly, each speech sample is independent since each patient was recorded only once. It is worth noting that the average age of the participants is 44.5 years. The recorded sessions encompass instances of participants delivering read speech, wherein they were specifically instructed to narrate a tale comprising approximately 10 sentences. During these recordings, participants were prompted to articulate and vocalize the content of the provided narrative. The audio signals were captured at a sampling frequency of 44.1 kHz with 16-bit quantization.

Importantly, each speech recording is associated with a HAMD score along with a mental health state diagnosis made by professional experts. It is important to highlight that approximately half of the individuals included in the database are healthy, whereas those diagnosed with depression represent various degrees of severity, distributed relatively evenly across the spectrum. Overall, the corpus includes 155.6 min of speech coming from depressed subjects and 167.3 min of speech coming from healthy ones. In addition, the dataset comes with a standardized experimental protocol, i.e., leave-one-subject-out cross-validation, allowing reliable comparison between contrasting approaches. The figures of merit are classification accuracy, sensitivity and specificity, root mean square error (RMSE), and mean absolute error (MAE). Detailed information regarding the dataset is available at [31].

### Parameterization of the Proposed Approach

The audio signals were normalized via DC-offset removal, while the features following the *z*-score standardization. During the extraction of the log-Mel spectrograms, we employed a window size of 30ms overlapped by 20ms, while the FFT length was set equal to 2048 and the number of bands 64.

Next, we applied regularization to the mel-scaled spectrograms through cepstral mean and variance normalization (CMVN), aiming to eliminate channel distortions and colorizations. CMVN has proven effective in minimizing variations in feature representation among speakers, as reported in previous studies [32].

### Analysis of the Results and Comparison with the State of the Art

This subsection presents the experimental results reached by the proposed approach and how they compare to the state of the art. Interestingly, we employed the same learned representation to address two different but related tasks, i.e., depression classification and severity assessment.

The results with respect to the classification task are provided in Table 1, while the highest values per figure of merit are emboldened. There, we see that the proposed approach significantly outperforms the state of the art in all considered figures of merit. More specifically, SNN reaches classification accuracy equal to 92.1%, while sensitivity and specificity are equal to 93.7% and 90.5%, respectively. On average the proposed solution outperforms the state of the art based on handcrafted features and SVM by 8.1%. Moreover, such an approach outperforms the results present in the literature, such as [33] where a method based on a long short term memory network achieves 84.3%, 81.8%, and 87% of classification accuracy, sensitivity, and specificity, respectively.

**Table 1:** Results achieved on the first downstream task by the proposed approach and the state of the art

Approach	Classification acc.	Sensitivity	Specificity
Proposed	**92.1±0.5%**	**93.7±0.3%**	**90.5±0.4%**
Contrasted [15]	84±1.6%	79±2.1%	89±1.8%

The highest recognition rates are emboldened, while standard deviation values over 50 iterations are included

It should be mentioned that the proposed approach offers considerable value in terms of both sensitivity and specificity, i.e., it demonstrates promising capabilities both in identifying depressed and healthy subjects. Such results become even more important given the fact that the contrasted approach employed a gender-depended modeling process, i.e., assumed *a-priori* availability of the gender information and built different models for male and female subjects. Overall, the SNN learns representations able to distinctively characterize the two classes and offer more than satisfactory classification capabilities.

**Fig. 2:**
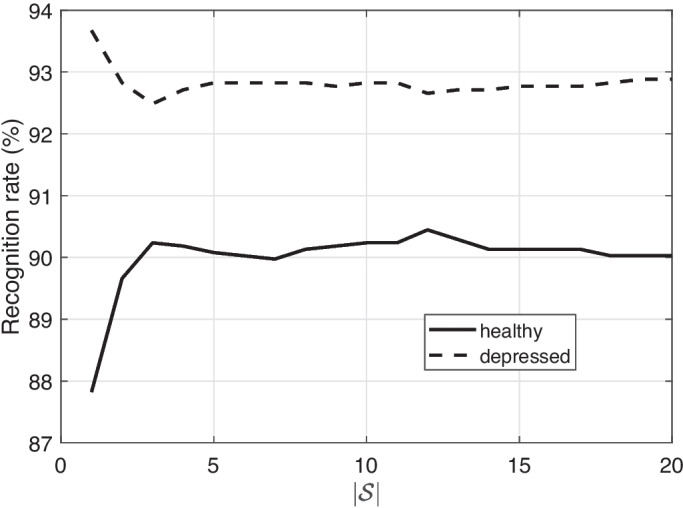
The recognition rate per class (healthy, depressed) when altering the size of the support set $\mathcal {S}$

Figure 2 demonstrates the way the recognition rate changes as the sizes of the support sets increase. More in detail, we see a rapid increase in identifying speech coming from healthy subjects during the first increment steps, while the highest rate is provided when $|\mathcal {S}^h|=12$. On the contrary, the rate when processing speech representing healthy subjects decreases after the first steps, while the highest rate is provided when $|\mathcal {S}^d|=1$. Both rates exhibit a relatively constant behavior after a certain size has been explored (approximately $\approx 5$). It should be mentioned that the optimum sizes of $|\mathcal {S}|$ were determined on a validation set equal to 10% of the training one. Such a difference in $|\mathcal {S}|$ may indicate that intra-class similarities are stronger in speech coming from healthy subjects depressed ones confirming the findings reported in [34].

Hence, the experience of depression varies considerably among distinct individuals, highlighting the diverse and unique ways in which people navigate and cope with this mental health condition. The nuanced nature of depression manifests uniquely in each subject, emphasizing the intricate interplay of factors that contribute to the diversity of individual experiences with this pervasive condition [34].

**Table 2:** Results achieved on the second downstream task by the proposed approach and the state of the art

Approach	RMSE	MAE
Proposed	**5**.**8**	**0**.**5**
[15]	8.2	6.3
[35]	9.54	n/a

The lowest RMSE and MAE scores (best performance) are emboldened

Moving to the second downstream task, i.e., prediction of the HAMD score, the results reached by the proposed approach (Sect. 5) and the contrasted one are presented in Table 2. There, we may observe that the proposed approach surpasses the state of the art. More specifically, the *k*-mediods algorithm using the learnt representation to compute the distance metric reaches RMSE of 5.8 and MAE of 0.5, while the best contrasted gender-depended SVM reached 8.2 and 6.3, respectively. The approach using x-vectors described in [35] reached an RMSE value of 9.54. We argue that the latent representation is able to offer relevant information strongly related to the HAMD score. Interestingly, the utilization of both male and female data in conjunction with the carefully crafted modeling scheme yields encouraging performance. The proposed approach, which involves incorporating information from both genders (*k*=24), has proven to be highly effective. In fact, it was observed that the composition of the nearest medoids always includes data coming from speakers of both genders. This diverse input not only enriches the learning process but also ensures that *k*-medoids is equipped to handle a broader range of scenarios and variations present in real-world applications.

**Fig. 3:**
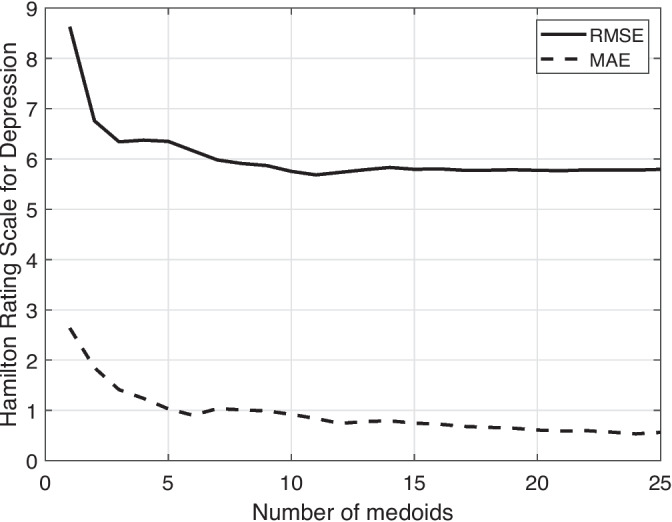
The RMSE and MAE values for HAMD score prediction while increasing the number of considered medoids

To additionally examine the results with respect to the second downstream task, Fig. 3 demonstrates the way RMSE and MAE change as the number of considered neighbor medoids increases. There, we observe a rapid decrease when the immediate neighborhood is considered, i.e., up to 5 closest medoids, while a relatively constant behavior follows. The minimum value for each metric is reached with a different *k*, (11 for RMSE and 24 for MAE), while their sum is minimized at *k*=24, thus comprises the chosen value. Overall, such behavior shows that considering data coming from similar speakers as judged by the learnt representation can be beneficial to predict the HAMD score. The implementation of the experimental pipeline is available at https://sites.google.com/site/stavrosntalampiras/home.

## Prediction Interpretation and Interaction with Medical Experts

This section emphasizes the significance of possessing a readily accessible interpretation of the entire AI pipeline, particularly in critical medical settings. Furthermore, we delve into the mechanisms through which the developed system can engage with medical professionals, fostering trust and effectively bridging the usability gap that often exists between AI-based tools and domain experts in the specific field.

**Fig. 4:**
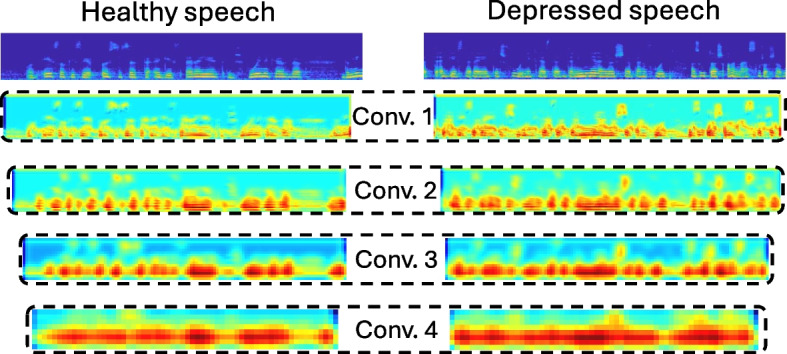
Representative activation maps with respect to the SNN’s convolutional layers when processing healthy and depressed speech segments

In the explanation stage, we analyze the processing of input spectrograms by each convolutional layer to pinpoint the areas that hold the most pertinent information for representation purposes. Figure 4 depicts the activation maps associated with the functioning of each convolutional layer as the SNN processes spectrograms generated from segments of healthy (left) and depressed (right) speech. Starting from the log-Mel spectrograms, it is evident that each layer systematically generates a condensed representation of the extracted features. This process aims at emphasizing specific regions that contain content conducive to optimizing the chosen distance function. The layers progressively distill the relevant information, prioritizing key aspects for the subsequent analysis. We observe that the SNN focuses on the speech content when processing both healthy and depressed speech segments, while the frequencies deemed relevant are within analogous bands. It is evident, especially in the initial convolutional layers, that the SNN concentrates on each formant independently from the rest when processing healthy speech, while such frequencies are collectively analyzed in the case of depressed speech. Given that a formant signifies the resonance occurring in the vocal tract and can be interpreted as the peak in the local spectrum, such finer processing is able to better represent the specific class. The specific information is particularly relevant in mental health assessment tasks as analytically discovered in related articles [36, 37]. Additionally, we observe that the SNN places significant emphasis on a low-frequency band when processing depressed segments, while it also considers the peripheral frequencies in the case of healthy segments. Furthermore, we note that the emphasis over time remains constant with respect to depressed speech, while there are clear pauses in the case of healthy speech. This is an outcome of the fact that depressed individuals tend to exhibit a reduced pace of speech [38].

Notably, the current framework offers medical professionals the chance to engage with it, gaining insights into the rationale behind each prediction. This interactive feature may aid experts in familiarizing themselves with such an AI-based tool, fostering trust a significant gap identified in the existing literature [13, 14]. More specifically, the proposed solution addresses three distinct types of queries:

- which time-frequency component influenced most the particular decision?
- which *a-priori* available speech segments present the strongest similarities to the processed one?
- which *a-priori* available speech segments present the strongest disssimilarities to the processed one?

When addressing the first query, the system provides a comprehensive response by: (1) showcasing the relevant activation map associated with the specific prediction (as in Fig. 4), (2) directing attention to regions of high relevance within the map, (3) pinpointing the corresponding content along the time-frequency axes, and (4) sonifying the identified content, allowing medical experts to exclusively listen to that particular segment.

Concerning queries 2 and 3, the proposed solution can readily provide clear responses, as such inquiries are directly related to the core functionalities of the entire system. As such, the identification of the most similar/dissimilar segments involves an initial examination of the support set $\mathcal {S}$, which includes the most characteristic speech recordings for each class. If the expert desires access to and wishes to listen to additional segments, the SNN can evaluate the distance between the unknown segment and the ones in $\mathcal {D}^{tr}$ to identify the most analogous (and disanalogous) ones, taking into account the *k*-medoids outputs as explained in Sect. 5. Crucially, the expert may have constant visibility of the similarity score, enabling assessment and providing insight into the system’s confidence in assessing both similar and dissimilar speech segments.

Such an interactive question-and-answer (Q&A) scheme can greatly facilitate medical experts in efficiently utilizing an AI-based solution. The overarching goal is to provide meaningful assistance to medical personnel. This multi-faceted approach ensures a thorough exploration and understanding of the factors influencing the final prediction.

## Conclusion

This work presented and thoroughly evaluated an SNN framework for speech-based mental health assessment. Importantly, such a framework learns a unique representation to address two diverse problems, i.e., the classification of depression and the quantification of its severity. Based on a standardized time-frequency representation, the system learns a summarized latent one via a SNN, which is then exploited to approach both tasks. After extensive experiments using a publicly available dataset following a reproducible and harmonized protocol, the strong effectiveness of the proposed framework was clearly demonstrated. Interestingly, the present method can easily expand and function in dynamic environments, allowing for the inclusion of further mental states as long as the relevant data becomes accessible, without the need for a full retraining process. Finally, a suitable Q&A-based interaction framework was outlined, capable of providing meaningful support to medical professionals throughout the diagnostic process. Such functionality has the potential to boost the acceptance of AI-driven solutions in the medical field, allowing experts to understand the reasoning behind each prediction. It not only helps complete the usability loop but also contributes to the education of young psychiatrists and medical personnel.

In the upcoming phases of the present research, we wish to investigate the next topics: (a) adjust the existing system to accommodate tasks with comparable requirements, (b) incorporate additional mental states, (c) investigate feature-level temporal integration methodologies (such as statistics, spectral moments, autoregressive models, etc.) considering the relatively slow changes in mental states over time, and (d) enhance the interpretability module’s functionalities, placing specific emphasis on user-friendliness and gaining acceptance among medical experts.

## Data Availability

We do not have distribution rights for the employed dataset.
